# From Cadavers to Soundwaves: The Evolution of Autopsy in Medical Diagnosis and the Rise of Point-of-Care Ultrasound

**DOI:** 10.7759/cureus.79684

**Published:** 2025-02-26

**Authors:** Guillermo Izquierdo-Pretel

**Affiliations:** 1 Hospital Medicine, Jackson Memorial Hospital, Miami, USA; 2 Internal Medicine, Florida International University, Herbert Wertheim College of Medicine, Miami, USA

**Keywords:** autopsy, clinical pathology, clinicopathological conference (cpc), diagnostic accuracy, evidence-based medicine (ebm), forensic medicine, medical error detection, point-of-care ultrasound (pocus), postmortem diagnosis, virtopsy (virtual autopsy)

## Abstract

This article aims to explore how closely point-of-care ultrasound (POCUS) aligns with final diagnoses and whether it could serve as a modern macroscopic alternative to autopsy. To address this question, a comprehensive literature review was conducted, examining the historical decline of autopsy, the influence of evidence-based medicine on diagnostic confidence, and the potential of POCUS to enhance clinical decision-making.

POCUS has demonstrated high accuracy in detecting major pathological conditions, particularly in critical care and emergency settings, and its use has now extended to primary care. However, its role as a "bedside autopsy" remains debated. While POCUS provides real-time, macroscopic assessment, it does not allow for histopathological analysis, limiting its ability to determine the definitive cause of death. Despite these limitations, integrating POCUS into clinical workflows may help reduce diagnostic discrepancies and serve as a practical alternative in settings where traditional autopsy is unavailable or underutilized.

Although POCUS cannot fully replace traditional autopsy, it offers a valuable tool for macroscopic postmortem assessment and may help bridge some of the diagnostic gaps created by declining autopsy rates. Incorporating POCUS findings into clinicopathological conferences and medical education could enhance diagnostic accuracy and reinforce evidence-based clinical practice. Further research is needed to refine its applications in postmortem diagnostics and establish its optimal role in forensic and hospital-based settings.

## Introduction and background

Autopsy, from the Greek autopsia ("to see with one’s own eyes"), has long been considered the gold standard for establishing cause of death, validating medical diagnoses, and advancing medical education. Since the foundational work of Giovanni Morgagni and Rudolf Virchow, autopsies have been essential for understanding disease processes and correlating clinical findings with postmortem pathology [1,2]. William Osler, a strong advocate of autopsy-based learning, performed thousands of autopsies to refine medical knowledge and enhance diagnostic accuracy [3].

Despite its historical significance, autopsy rates have declined dramatically, from over 50% in the mid-20th century to less than 5% today [4,5]. This decline is attributed to several factors, including the advancement of imaging technologies such as computed tomography (CT), magnetic resonance imaging (MRI), and ultrasound (US), as well as legal and ethical considerations regarding informed consent and the financial burden associated with autopsy procedures [6,7]. However, studies continue to show that missed diagnoses remain common, with autopsies revealing major diagnostic discrepancies in 10-40% of cases [8].

The reduction of autopsy-based learning has also contributed to increased diagnostic uncertainty. Clinicopathological conferences (CPCs), once a core aspect of medical education, are now rare, limiting opportunities for clinicians to compare clinical impressions with actual pathology findings [9].

In response to these challenges, point-of-care ultrasound (POCUS) has emerged as a rapid, non-invasive diagnostic tool with the potential to bridge this gap. Given its ability to provide real-time insights into critical conditions such as cardiac arrest, shock, and internal bleeding, some experts propose that POCUS could serve as a “living autopsy,” offering immediate bedside diagnostic guidance [10,11].

This article explores the historical importance of autopsy, the factors leading to its decline, and the potential role of POCUS as an adjunctive tool to enhance clinical decision-making and patient care.

## Review

Methods

A comprehensive narrative review was conducted to explore the historical decline of autopsy, the role of evidence-based medicine in diagnostic accuracy, and the potential application of POCUS as a postmortem assessment tool. This allows for an open interpretation of the literature, providing a broader perspective on the topic, as well as a judgment-based selection of studies, ensuring that key articles with significant conceptual contributions were included, rather than being limited by rigid inclusion criteria. Thus, the concept-driven organization allows this review to be structured around relevant themes such as diagnostic accuracy, medical education, and the evolving role of POCUS in postmortem assessment.

This approach was deemed appropriate in comparison to a systematic review; thus, it spotlights the interdisciplinary nature of the topic, which involves aspects of radiology, pathology, medical education, and clinical decision-making. The review followed a structured approach to ensure comprehensiveness and reproducibility.

Search strategy

The search included peer-reviewed journal articles, systematic reviews, and relevant clinical guidelines published in English. Gray literature (conference proceedings and reports) was not included to maintain source reliability. A systematic literature search was conducted for studies published between January 2000 and April 2024 in the following electronic databases: PubMed, Scopus, Web of Science, and Google Scholar. 

Search terms

The search was conducted using combinations of the following keywords and Boolean operators:

("Autopsy" OR "postmortem examination" OR "forensic pathology") AND ("POCUS" OR "Point-of-Care Ultrasound" OR "bedside ultrasound") AND ("diagnostic accuracy" OR "medical error detection" OR "postmortem imaging") AND ("clinicopathological conferences" OR "medical education").

Inclusion and exclusion criteria

The inclusion and exclusion criteria for the studies to be included in this work are listed in Table 1.

**Table 1:** Inclusion and exclusion criteria, detailing the standards used in the search engine. POCUS, point-of-care ultrasound.

Inclusion criteria	Exclusion criteria
Published in peer-reviewed journals and discussed the impact of autopsy-based learning on medical education	Studies focused solely on traditional imaging (CT/MRI) without POCUS data
Addressed the role of autopsy, postmortem imaging, or POCUS in diagnostic accuracy	Articles lacking original data (opinion pieces, letters to the editor)
Contained comparative data on autopsy vs. POCUS	Studies not available in English

Studies prior to 2000 were excluded unless they were landmark papers on the historical role of autopsy or the evolution of medical education. This period ensures relevance and methodological rigor while allowing for a historical comparison where necessary. To ensure comprehensiveness and relevance, the period for the literature review was chosen from 2000 to 2024, covering recent advancements in POCUS, autopsy practices, and medical education while still considering historical perspectives.

Historical evolution of autopsy

Autopsy has played a crucial role in medical learning and the advancement of diagnostic accuracy for centuries. Early anatomists and physicians, such as Giovanni Morgagni (1682-1771) and Xavier Bichat (1771-1802), pioneered autopsy-based investigations to correlate clinical findings with postmortem pathology. Their work laid the foundation for modern pathology and disease classification [1-3]. In the 19th century, Karl Rokitansky (1804-1878) performed over 30,000 autopsies, refining systematic postmortem examination methods still in use today [4]. Rudolf Virchow (1821-1902) later revolutionized pathology by introducing the concept that disease originates from cellular abnormalities, a principle that remains fundamental to modern medicine [9]. Sir William Osler (1849-1919), a strong proponent of autopsy-based learning, integrated postmortem studies into medical education, reinforcing its value in developing clinical acumen [3,4].

The decline of autopsies in the 21st century

Despite its historical significance, clinical autopsy rates have sharply declined, from 50% in the mid-20th century to less than 5% today [5]. This decline is attributed to several factors, including the rise of imaging technologies (CT, MRI, and US), which falsely suggest that autopsies are no longer necessary, legal and ethical concerns, including consent issues from families, and financial constraints, as many healthcare systems do not reimburse autopsy procedures [12,13]. However, studies continue to demonstrate that diagnostic errors remain common, with autopsies revealing major discrepancies in 10-40% of cases [11]. A meta-analysis found that nearly one in four deaths involved a significant missed diagnosis that, if recognized earlier, could have altered management or outcomes [14].

The diagnostic dilemma: How can clinicians improve accuracy?

With the decline in autopsy-based learning, clinicians now face increasing diagnostic uncertainty despite advancements in imaging and laboratory testing. Studies suggest that overreliance on imaging can lead to false-positive and false-negative findings, increasing diagnostic errors [15,16]. Clinicians are losing exposure to direct pathology correlations, as autopsies are no longer standard in medical training [9,17]. CPCs, once a core aspect of diagnostic education, are now rare, reducing opportunities to recognize missed diagnoses [2,18]. Given this shift, alternative approaches to improving diagnostic accuracy are essential.

Can POCUS be the "new autopsy"?

While POCUS cannot replace traditional autopsy from a scientific standpoint, it presents a practical adjunct in specific cases where autopsy is unavailable or declined. As a non-invasive, real-time imaging tool, POCUS may help partially bridge the diagnostic gap created by declining autopsy rates. Unlike CT or MRI, it provides immediate bedside insights into critical conditions such as cardiac tamponade, pneumothorax, and shock states, offering clinicians valuable macroscopic assessment when a full postmortem examination is not feasible [19,20].

For example, POCUS is widely used in cardiac arrest settings, yet its limitations must be acknowledged, particularly regarding operator dependency and image quality variability. Accurate interpretation requires training, and image acquisition can be challenging due to factors such as body habitus, fluid accumulation, and postmortem gas artifacts [6]. In postmortem applications, while POCUS does not replace traditional autopsy, it can provide rapid macroscopic insights, particularly in cardiovascular pathologies, as well as in age estimation or forensic examinations of living victims of violence [21].

Studies suggest that while POCUS cannot fully replace traditional autopsy, it offers rapid, macroscopic postmortem assessments that can help clinicians identify major pathological conditions [22,23]. Additionally, in emergency and critical care settings, POCUS has shown high diagnostic accuracy in detecting life-threatening abnormalities and has even been proposed as an adjunct in forensic medicine [24]. However, it lacks histopathological capabilities, making it insufficient for definitive cause-of-death determination [25]. In forensic settings, traditional autopsy remains essential due to its ability to provide legal admissibility, full-body examination, toxicology testing, and histopathological confirmation. While POCUS may serve as a triage tool or assist in cases where autopsy is unavailable or declined, it should be viewed as a complementary rather than a replacement tool. A multimodal approach that integrates POCUS with postmortem imaging (CT/MRI) and traditional autopsy can enhance diagnostic accuracy in both clinical and forensic medicine.

Hence, it must be reiterated that POCUS offers a non-invasive and rapid alternative for postmortem assessment, and its cost-effectiveness and implementation feasibility vary across healthcare systems. Compared to traditional autopsy, POCUS requires lower operational costs, fewer resources, and no specialized pathology infrastructure, making it a potentially viable option in resource-limited settings [26]. However, the initial investment in US equipment and clinician training presents a barrier to widespread adoption [27]. Despite this limitation, integrating POCUS into postmortem investigations and CPCs can help bridge some of the diagnostic gaps left by declining autopsy rates [11], and thus, this balanced integration could improve medical education, forensic investigations, and diagnostic precision, ensuring that the declining autopsy rates do not compromise postmortem diagnostics.

Now, the accuracy of POCUS will depend on operator expertise, necessitating specialized training programs for clinicians. Unlike autopsy, which follows standardized forensic and clinical protocols, POCUS lacks universal postmortem guidelines, raising concerns about inter-operator variability and diagnostic reproducibility. Thus, the use of POCUS in postmortem investigations is not universally standardized, and its legal admissibility in forensic cases remains debated. Some healthcare systems may require institutional approvals or forensic validation before POCUS can be incorporated into death investigations or clinical auditing processes [11,25].

Reflections and perspectives 

Although POCUS offers a valuable adjunct to postmortem assessment, it is not a substitute for traditional autopsy. The decline in autopsy rates has undoubtedly contributed to increased diagnostic uncertainty, raising the question of whether a revitalization of autopsy practices should be encouraged. Given its ability to provide definitive histopathological analysis, autopsy remains an irreplaceable tool in medical education, forensic investigations, and quality assurance.

However, in cases where autopsy is unavailable, declined by families, or not feasible due to institutional limitations, POCUS presents a practical alternative for immediate macroscopic assessment. Its advantages -- real-time imaging, non-invasiveness, and bedside applicability -- can help enhance clinical decision-making, reduce diagnostic errors, and support evidence-based practice.

Rather than positioning POCUS as a replacement for autopsy, a balanced approach should be encouraged. This includes integrating POCUS with postmortem imaging modalities, reinforcing the role of CPCs, and advocating for increased autopsy utilization where possible. Such an approach would maximize diagnostic accuracy, improve medical education, and bridge the knowledge gap left by the declining autopsy.

In particular, POCUS has demonstrated utility in identifying major conditions traditionally confirmed at autopsy, such as cardiac arrest-related complications, sepsis-induced organ failure, and blunt trauma injuries. Table 2 outlines a comparison between autopsy-confirmed findings and POCUS-based assessments for these conditions. Further research is needed to assess the extent to which POCUS can serve as a practical alternative where traditional autopsy is unavailable or underutilized.

**Table 2:** Comparison of autopsy findings and POCUS as a diagnostic alternative eFAST, extended focused assessment with sonography in trauma; DIC, disseminated intravascular coagulation; ICP, intracranial pressure; POCUS, point-of-care ultrasound.

Clinical condition	Autopsy findings	POCUS alternative
Cardiac arrest	Pulmonary embolism, tamponade	Right heart strain, pericardial effusion [28,29]
Sepsis and shock	Multi-organ failure, DIC	Inferior vena cava collapsibility [30]
Blunt trauma	Solid organ laceration, hemoperitoneum	eFAST for intra-abdominal bleeding [31]
Neurological events	Ischemic infarct, herniation	Optic nerve sheath diameter for ICP [32]

This review follows a narrative literature review approach, aiming to provide a conceptual comparison between autopsy and POCUS findings rather than a quantitative meta-analysis of diagnostic performance. Given this approach, statistical metrics such as sensitivity, specificity, and accuracy rates were not included. Instead, relevant studies discussing these metrics are cited where appropriate to provide context for readers. 

## Conclusions

While POCUS cannot fully replace traditional autopsy, it serves as a valuable macroscopic postmortem assessment tool, helping to bridge diagnostic gaps left by declining autopsy rates. Its key advantages include real-time imaging, non-invasiveness, and bedside applicability, making it particularly useful in settings where autopsy is unavailable or underutilized. However, POCUS lacks histopathological analysis and may not detect microscopic or systemic pathologies, limiting its ability to determine the definitive cause of death. Despite these limitations, integrating POCUS findings into CPCs and medical education could enhance diagnostic accuracy and reinforce evidence-based practice. Further research is needed to refine its role in postmortem diagnostics and determine its optimal application in forensic and hospital settings.

Although technological advancements continue to emerge, autopsy remains the most definitive diagnostic tool for confirming causes of death and refining medical knowledge. However, its decline has created a gap in medical education and clinical practice, increasing diagnostic uncertainty.

POCUS may serve as a partial replacement for traditional autopsy, offering real-time, non-invasive insights that improve bedside diagnosis. While POCUS cannot fully replace postmortem histopathological analysis, it provides clinicians with an immediate assessment of critical conditions, potentially reducing diagnostic errors. By integrating POCUS, postmortem imaging, and revived CPCs into medical training, physicians can enhance their diagnostic skills and mitigate the knowledge gap left by the declining use of autopsy in modern medicine.

## References

[1] Goldman L, Sayson R, Robbins S, Cohn LH, Bettmann M, Weisberg M (1983). The value of the autopsy in three medical eras. N Engl J Med.

[2] Zampieri F, Rizzo S, Thiene G, Basso C (2015). The clinico-pathological conference, based upon Giovanni Battista Morgagni's legacy, remains of fundamental importance even in the era of the vanishing autopsy. Virchows Arch.

[3] Rodin AE (1973). Osler's autopsies: Their nature and utilization. Med Hist.

[4] Roberts CS (2009). Osler under 40. Proc (Bayl Univ Med Cent).

[5] De Bruin JSA (2021). Is Medical Postmortem Practice Dying? An Audit on Postmortems in the Department of Anatomical Pathology, University of the Witwatersrand, Johannesburg, South Africa. Research Report Submitted to the Faculty of Health Sciences, University of the
Witwatersrand, Johannesburg. https://wiredspace.wits.ac.za/server/api/core/bitstreams/97b8c523-11cc-4372-85fa-f32bb2368b08/content.

[6] Dehner LP (2010). The medical autopsy: Past, present, and dubious future. Mo Med.

[7] van den Tweel JG, Wittekind C (2016). The medical autopsy as quality assurance tool in clinical medicine: Dreams and realities. Virchows Arch.

[8] Shojania KG, Burton EC, McDonald KM, Goldman L (2003). Changes in rates of autopsy-detected diagnostic errors over time: A systematic review. JAMA.

[9] Burton JL, Underwood J (2007). Clinical, educational, and epidemiological value of autopsy. Lancet.

[10] Magon F, Longhitano Y, Savioli G (2024). Point-of-care ultrasound (POCUS) in adult cardiac arrest: Clinical review. Diagnostics (Basel).

[11] Eriksson A, Gustafsson T, Höistad M, Hultcrantz M, Jacobson S, Mejare I, Persson A (2017). Diagnostic accuracy of postmortem imaging vs autopsy-A systematic review. Eur J Radiol.

[12] Shojania KG, Burton EC, McDonald KM, Goldman L (2002). Autopsy as an outcome and performance measure: Summary. AHRQ Evidence Report Summaries.

[13] Xiao J, Krueger GR, Buja LM, Covinsky M (2009). The impact of declining clinical autopsy: Need for revised healthcare policy. Am J Med Sci.

[14] Winters B, Custer J, Galvagno SM Jr (2012). Diagnostic errors in the intensive care unit: A systematic review of autopsy studies. BMJ Qual Saf.

[15] Stevanovic G, Tucakovic G, Dotlic R, Kanjuh V (1986). Correlation of clinical diagnoses with autopsy findings: A retrospective study of 2,145 consecutive autopsies. Hum Pathol.

[16] Sandritter W, Staeudinger M, Drexler H (1980). Autopsy and clinical diagnosis. Pathol Res Pract.

[17] Horowitz RE, Naritoku WY (2007). The autopsy as a performance measure and teaching tool. Hum Pathol.

[18] Feinstein AR, Niebyl JR (1971). Changes in the diagnostic process during 40 years of clinicopathologic conferences. Arch Intern Med.

[19] Goldsmith AJ, Shokoohi H, Loesche M, Patel RC, Kimberly H, Liteplo A (2020). Point-of-care ultrasound in morbidity and mortality cases in emergency medicine: Who benefits the most?. West J Emerg Med.

[20] Casado-Suela MA, Cuevas-Tascón G, Cabezas Quintario MA (2023). Could we consider ultrasound-guided minimally invasive autopsy as a part of POCUS?. J Ultrasound Med.

[21] Auerbach A, Lee T, Hubbard C (2024). Diagnostic errors in hospitalized adults who died or were transferred to intensive care. JAMA Intern Med.

[22] Shaban EE, Yigit Y, Alkahlout B (2025). Enhancing clinical outcomes: Point of care ultrasound in the precision diagnosis and management of abdominal aortic aneurysms in emergency medicine: A systematic review and meta-analysis. J Clin Ultrasound.

[23] Yacoub S (2024). The use of point-of-care ultrasound for out-of-hospital cardiac arrest management: A review of the literature. Curr Emerg Hosp Med Rep.

[24] Ravikanth R (2021). Diagnostic accuracy and prognostic significance of point-of-care ultrasound (POCUS) for traumatic cervical spine in emergency care setting: A comparison of clinical outcomes between POCUS and computed tomography on a cohort of 284 cases and review of literature. J Craniovertebr Junction Spine.

[25] Reynolds JC, Nicholson T, O'Neil B, Drennan IR, Issa M, Welsford M (2022). Diagnostic test accuracy of point-of-care ultrasound during cardiopulmonary resuscitation to indicate the etiology of cardiac arrest: A systematic review. Resuscitation.

[26] Hannula O (2023). Point-of-care ultrasound in urgent and emergency settings in Finland: Deep venous thrombosis in primary care and self-assessed ultrasound skills of physicians. University of Eastern Finland Repository. https://erepo.uef.fi/bitstreams/57fed794-d4ac-4ba9-a57d-a0bfd4f508e3/download.

[27] Ilieșiu AM, Hodorogea AS, Balahura AM, Bădilă E (2022). Non-invasive assessment of congestion by cardiovascular and pulmonary ultrasound and biomarkers in heart failure. Diagnostics (Basel).

[28] Amagasa S, Kashiura M, Moriya T (2019). Relationship between institutional case volume and one-month survival among cases of paediatric out-of-hospital cardiac arrest. Resuscitation.

[29] Ventorp S, Wagner H, Hardig B (2023). Does point-of-care ultrasound improve survival when used during cardiac arrest? A systematic review and meta-analysis. Med Res Arch.

[30] Kaptein MJ, Kaptein EM (2021). Inferior vena cava collapsibility index: Clinical validation and application for assessment of relative intravascular volume. Adv Chronic Kidney Dis.

[31] Netherton S, Milenkovic V, Taylor M, Davis PJ (2019). Diagnostic accuracy of eFAST in the trauma patient: A systematic review and meta-analysis. CJEM.

[32] Yic CD, Pontet J, Mercado M, Muñoz M, Biestro A (2023). Ultrasonographic measurement of the optic nerve sheath diameter to detect intracranial hypertension: An observational study. Ultrasound J.

